# Cascade screening for familial hypercholesterolemia-identification of the C308Y mutation in multiple family members and relatives for the first time in mainland China

**DOI:** 10.1186/s12881-019-0901-0

**Published:** 2019-11-09

**Authors:** Weirong Jin, Qiuwang Zhang, Bei Wang, Lili Pan, Hongyou Qin, Daying Yang, Xiangqun Zhou, Yongcai Du, Ling Lin, Michael J. Kutryk

**Affiliations:** 1 Shanghai Human Genome Center, Shanghai, China; 2 Division of Cardiology, Keenan Research Center for Biomedical Science, St. Michael’s Hospital, University of Toronto, Toronto, Ontario Canada; 3 Department of Cardiology, the Third People’s Hospital of Hainan Province, 1154 Jiefang Road, Sanya, 572000 Hainan Province China

**Keywords:** Familial hypercholesterolemia, Cascade screening, C308Y mutation, Low density lipoprotein-cholesterol, Low density lipoprotein receptor, DNA sequencing

## Abstract

**Background:**

Familial hypercholesterolemia (FH), an autosomal dominant genetic disorder, is underdiagnosed and undertreated. The majority of FH cases are caused by low density lipoprotein receptor (*LDL-R*) gene mutations. The C308Y mutation in *LDL-R* results in approximately 70% loss of LDL-R activity, leading to the elevation of low density lipoprotein-cholesterol (LDL-C) and an increased risk of premature coronary heart disease (CHD). The aim of this study was to identify FH cases by cascade screening in family members and relatives of a 37-year old male with premature CHD and hypercholesterolemia.

**Methods:**

Clinical exam, blood lipid profiling and genomic DNA sequencing of all exons of *LDL-R* were performed for the proband and his 14 family members and relatives. FH diagnosis was carried out using the Dutch Lipid Clinic Network (DLCN) criteria.

**Results:**

Lipid profiling showed that 9 individuals, including the proband, had hypercholesterolemia. All these 9 subjects had a G > A substitution at nucleotide 986 in exon 7 resulting in the C308Y mutation as determined by DNA sequencing, and all those carrying the mutation were diagnosed as having definite FH under the DLCN criteria. However, most (7/9) did not have suggestive clinical manifestations of CHD.

**Conclusions:**

The C308Y mutation was discovered in multiple family members and relatives for the first time in mainland China. Cascade screening is key for the confirmatory diagnosis of FH. Our hypothesis that the C308Y is a common variant in the population of Southern China origin warrants further validation by screening for the C308Y mutation in a large population.

## Background

Familial hypercholesterolemia (FH) is an autosomal dominant genetic disorder characterized by elevated levels of low density lipoprotein-cholesterol (LDL-C), tendon xanthomas and an increased risk of premature coronary heart disease (CHD) [1]. It is caused by mutations of genes encoding proteins that are responsible for the metabolic clearance of LDL-C. To date, several genes whose mutations linked to FH have been identified, which include the low density lipoprotein receptor (*LDL-R*) gene, the apolipoprotein B gene, the proprotein convertase subtilisin/kexin type 9 gene and the LDL receptor adapter protein 1 gene [2]. Most cases (60–80%) of FH are caused by mutations in the *LDL-R* gene [3].

Homozygous *LDL-R* mutations are rare (1/1 million) [4], but the prevalence of heterozygous FH is high. A recent meta-analysis of 19 studies covering almost 2.5 million individuals has revealed that the prevalence of heterozygous FH is 1/250 in general population [5], which doubles a previous estimation of 1/500 [4]. Early diagnosis of FH is critical for the initiation of effective pharmacological treatment that can lower LDL-C concentrations and reduce the risk of CHD [6].

The *LDL-R* is a ~ 45 kb gene containing 18 exons [7]. Although previous data suggest that the majority of *LDL-R* mutations occur in exons 3 and 4, the current understanding is that mutations causing FH are distributed throughout the entire gene including promotor, intronic and 3′-untranslated regions [8]. The updated *LDL-R* database of University College London (now the Leiden Open Source Variation Database, LOVD) has documented a total of 1959 *LDL-R* variants (https://databases.lovd.nl/shared/genes/LDLR). Nevertheless, the geographical- or population-specific fingerprints of *LDL-R* mutations have been noted [9–14]. Therefore, identification of predominant mutations in a certain region or population could direct strategies for molecular detecting. For example, when a small number of mutations predominate, molecular tests can be designed to identify these specific variant alleles, making testing more cost-effective and time saving.

There is a strong consensus on the facts that a diagnosis of FH can be made on the basis of clinical criteria (elevated LDL-C levels, clinical history of premature cardiovascular disease, family history of FH and physical examination findings such as tendon xanthomas) and/or DNA testing; and that when a pathogenic FH mutation is identified a definite diagnosis can be made [15–17]. A recent systematic review of guidelines on genetic testing and management of FH has found that most guidelines recommend the use of DNA testing for cascade screening [18]. DNA testing offers definitive diagnoses, which ensures prompt treatment that can significantly reduce the risk of cardiovascular disease. Additionally, DNA testing is capable of distinguishing heterozygous from homozygous FH, which can direct patient care, as more aggressive therapeutic intervention is required for those with homozygous FH.

A recent systemic review of *LDL-R* mutations in Chinese population revealed regional clustering of *LDL-R* mutations, e.g., A606T, D601Y and W462X are the primary mutations found in mainland China while C308Y and H562Y predominate in Taiwan province and Hong Kong [14]. To date, only one C308Y case has been discovered in mainland China [19]. Here we reported the identification of multiple family members and relatives of a family with the C308Y mutation in Hainan Province, China. Physical examinations, lipid profiling, DNA sequencing and the DLCN criteria were used to investigate the index patient and his 14 family members and relatives.

## Methods

### Ethical approval

This study was approved by the Research Review Committee of the Third People’s Hospital of Hainan Province. Written informed consent to participating in the study was obtained from all subjects.

### Study subjects

A 37-year old male (proband) with severe early onset coronary artery disease and his 14 family members and relatives were recruited for this study.

### Blood lipid profiling

Blood lipid testing was routinely performed at the department of clinical laboratory, the Third People‘s Hospital of Hainan Province.

### Measurement of Achilles tendon thickness

Achilles tendon thickness was measured using a standardized digital radiography method as we described elsewhere [20]. Patients with an Achilles tendon thickness of ≥9 mm was taken as tendon thickening [21].

### *LDL-R* mutation analysis

Genomic DNA was extracted from 5 to 10 ml peripheral blood using the Blood Genomic DNA Extraction Kit (Tiangen Biotech Co., Ltd., Beijing, China) according to the manufacturer’s instructions, and quantified using a ND-1000 spectrophotometer (Thermo Fisher Scientific China Co., Ltd., Shanghai, China). PCR was done to amplify each of 18 exons of *LDL-R* using 50 ng genomic DNA as template. All primers (Table 1) were designed based on the *LDL-R* sequence (NCBI Reference Sequence #: NG_009060.1). PCR was performed as follows: stage 1, 95 °C for 5 min; stage 2, 95 °C for 30s, 55 °C for 40s and 72 °C for 40s; and stage 2 was repeated for 35 cycles. Afterwards, the PCR product was purified using the QIAquick PCR Purification Kit (Qiagen China Co., Ltd., Shanghai, China) and sequenced using the Sanger method. DNA sequencing results were analyzed using the software Sequencher 5.0 (Gene Codes Corp., Ann Arbor, MI, USA).

**Table 1:** PCR primers for amplifications of all coding exons of *LDL-R*

Exon	Forward	Reverse	Product size
1	5′-GTGGGAATCAGAGCTTCACG-3’	5′-GGATGGAGTGATTATTTGTA-3’	410 bp
2	5′-GATTCTGGCGTTGAGAGACC-3’	5′-AGCGGATCACTTGAGACCAG-3’	271 bp
3	5′-GGTCTTTCCTTTGAGTGACAGT-3’	5′-CCACTTTGTAATGCCTCCTGG-3’	353 bp
4	5′-TAGAATGGGCTGGTGTTGGG-3’	5′-CCAGGGACAGGTGATAGGAC-3’	496 bp
5	5′-GTACAGACACAGGCTGGTCT-3’	5′-CCCTCTGGCTTCACAAATCA-3’	372 bp
6	5′-CACACCTGACCTTCCTCCTT-3’	5′-TCCCAAAACCCTACAGCACT-3’	245 bp
7	5′-AGTGACCAGTCTGCATCCC-3’	5′-GGTTGCCATGTCAGGAAGC-3’	211 bp
8	5′-CCAAGCCTCTTTCTCTCTCTTC-3’	5′-GGGATATGAGTCTGTGCAAAGT-3’	250 bp
9	5′-GCACTCTTGGTTCCATCGAC-3’	5′-GAGCCCTCATCTCACCTGC-3’	285 bp
10	5′-CAGGTGAGATGAGGGCTCC-3’	5′-TCCTTCCTGCTCCCTCCAT-3’	333 bp
11	5′-CAGCTATTCTCTGTCCTCCCA-3’	5′-GTGACAGACCAAGACCTCATC-3’	306 bp
12	5′-CAGCACGTGACCTCTCCTTA-3’	5′-AGTCTGTGTCTATCCGCCAC-3’	229 bp
13	5′-CCTGTGTCTCATCCCAGTGT-3’	5′-CAAGGAGGTTTCAAGGTTGGG-3’	244 bp
14	5′-GCTGATGATCTCGTTCCTGC-3’	5′-GACACAGGACGCAGAAACAA-3’	232 bp
15	5′-CACGTGGCACTCAGAAGAC-3’	5′-GGACTCCATCTCGTGACCAA-3’	296 bp
16	5′-GGCCTCACTCTTGCTTCTCT-3’	5′-CCATCTGACCCCTTAGCTGT-3’	299 bp
17	5′-GTTTTCACTCCAGCCACGG-3’	5′-TGGCTTTCTAGAGAGGGTCAC-3’	258 bp
18	5′-TGTTTACCATTTGTTGGCAG-3’	5′-TTGGTCTTCTCTGTCTTTG-3’	140 bp

### Diagnosis of FH

The Dutch Lipid Clinic Network (DLCN) criteria were used for the diagnosis of FH. This diagnostic algorithm uses scores created based on the examination results from following 5 areas: family history, clinical history, physical examination, LDL-C levels and molecular genetic testing [17]. According to the score, diagnosis is made as follows: a score of > 8 points for a ‘definite FH’; 6 to 8 points for ‘probable FH’; 3 to 5 points for ‘possible FH’; and 0 to 2 points for ‘unlikely FH’.

## Results

### Study subjects

The proband presented to our hospital with a two week history of crescendo angina. Coronary angiography showed severe 3-vessel coronary artery disease. Achilles tendon thickness measured 13 mm bilaterally. The proband’s father had a history of CHD, while clinical manifestations suggestive for CHD were not present in other family members and relatives. Of the 5 individuals who consented to measurement of Achilles tendon, 4 had Achilles tendon thickness ≥ 9 mm. The demographics and clinical characteristics of all subjects are described in Table 2.

**Table 2:** Demographics and clinical characteristics of all subjects

Subject No	Relationship with the proband	Gender	Age (Years)	BMI	Smoking (Years)	TX	ATT (mm) L, R	BP (mmHg)
II-9	Proband	M	30’s-40’s	24.6	> 20	Yes	13, 13	150/60
I-3	Father	M	60’s-70’s	24.2	> 30	No	–	150/98
I-4	Mother	F	60’s-70’s	19.9	–	No	–	130/80
III-2	Son	M	10’s-20’s	14.5	–	No	–	92/50
II-6	Sister 1	F	40’s-50’s	17.5	–	No	–	140/80
II-7	Sister 2	F	40’s-50’s	26.0	–	No	7, 7	130/90
II-8	Sister 3	F	30’s-40’s	18.6	–	Yes	9, 9	122/80
II-10	Brother	M	20’s-30’s	25.3	–	Yes	9, 9	122/88
II-11	Sister 4	F	30’s-40’s	28.4	–	No	–	140/90
II-1	Cousin 1	F	50’s-60’s	23.4	–	No	–	140/95
II-2	Cousin 2	F	50’s-60’s	19.5	–	No	–	150/90
II-3	Cousin 3	F	40’s-50’s	20.4	> 20	Yes	9, 10	130/90
II-4	Cousin 4	M	40’s-50’s	20.8	> 20	No	–	160/100
II-5	Cousin 5	M	40’s-50’s	16.4	–	No	–	110/70
III-1	Nephew	M	20’s-30’s	18.6	–	No	–	140/70

Only 5 members had Achilles tendon thickness measured while the rest declined the measurement

*BMI* body mass index, *TX* tendon xanthoma, *ATT* Achilles tendon thickness, *L* left, *R* right, *BP* blood pressure

### Blood lipid profiling

Results of blood lipid testing for all subjects are shown in Table 3. According to the diagnostic criteria for hypercholesterolemia set in the “Chinese Guidelines on Prevention and Treatment of Dyslipidemia in Adults”, i.e., LDL-C ≥ 4.14 mmol/L [22], 9 out of 15 subjects had hypercholesterolemia.

**Table 3:** Blood lipid profiling and mutation results of all subjects

Subject No	Mutation	TG levels (mmol/L)	TC levels (mmol/L)	LDL-C levels (mmol/L)	HDL-C levels (mmol/L)	HCL
II-9	Yes	2.80	9.96	7.44	1.4	Yes
I-3	Yes	1.07	8.69	6.56	2.1	Yes
I-4	No	2.17	4.86	2.69	1.3	No
III-2	Yes	0.90	8.05	6.19	1.5	Yes
II-6	No	2.52	5.05	2.74	1.3	No
II-7	Yes	2.98	10.96	8.27	1.5	Yes
II-8	Yes	0.66	7.45	5.39	1.8	Yes
II-10	Yes	1.62	10.14	7.89	1.6	Yes
II-11	No	1.70	6.43	3.25	2.5	No
II-1	Yes	2.24	11.20	7.60	2.7	Yes
II-2	No	2.45	5.53	2.95	1.6	No
II-3	Yes	1.13	9.08	6.83	1.8	Yes
II-4	No	6.22	8.06	3.37	2.2	No
II-5	Yes	0.82	9.96	6.73	2.9	Yes
III-1	No	0.57	4.08	2.15	1.7	No

*TG* triglyceride, *TC* total cholesterol, *LDL-C* low density lipoprotein-cholesterol, *HDL-C* high density lipoprotein-cholesterol, *HCL* hypercholesterolemia

### *LDL-R* sequencing results

**Fig. 1:**
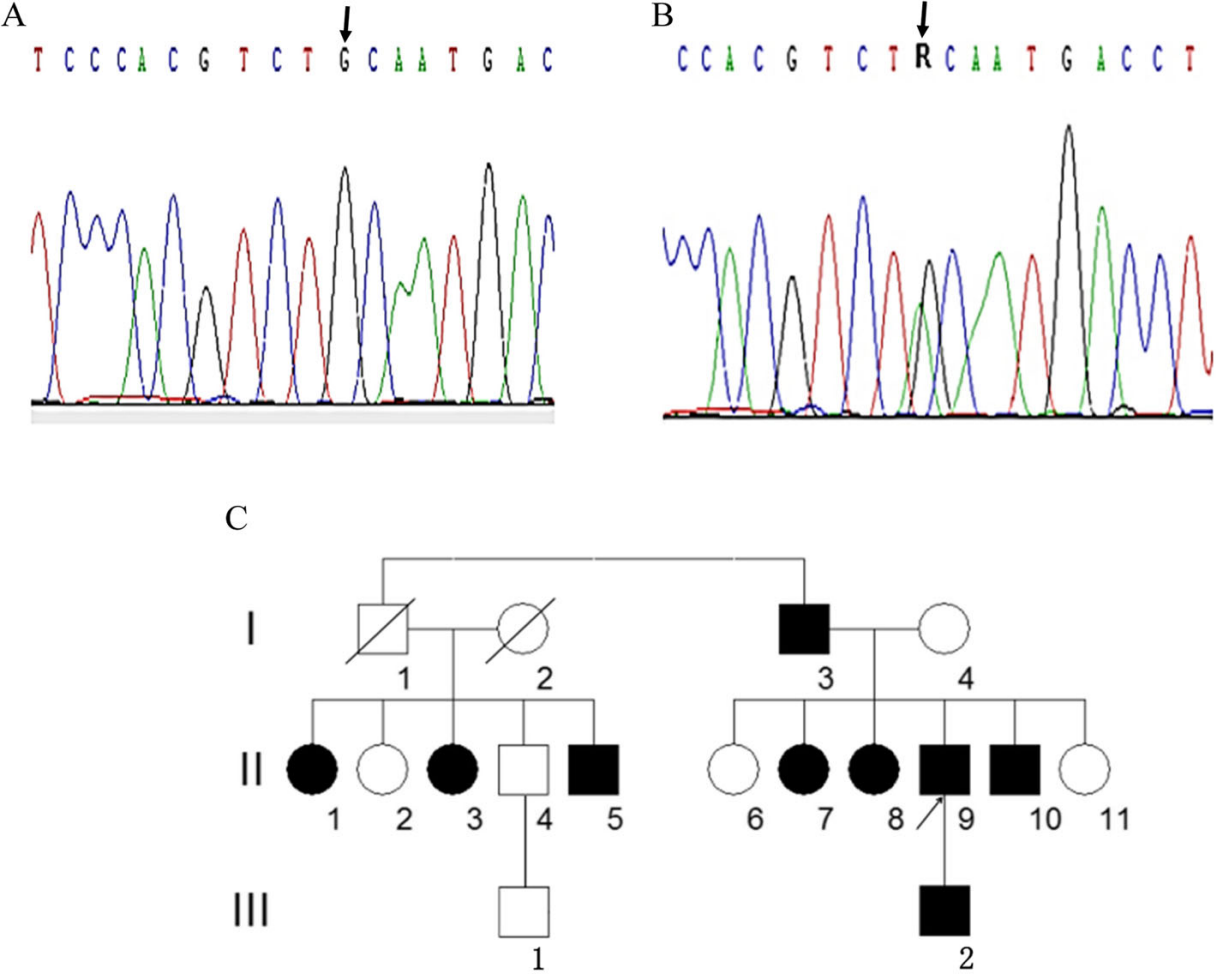
DNA sequencing results and the pedigree chart. Shown here are the wild-type (panel **a**) and the C308Y mutation (panel **b**) DNA sequencing histograms. The nucleotide G at position 923 of exon 7 in the wildtype (arrow in panel **a**) was mutated to the nucleotide A (arrow in panel **b**) in the heterozygote. According to the DNA sequencing results, a pedigree chart was established (panel **c**). Unfilled squares are healthy males and filled squares are mutant males; unfilled circles indicate healthy females and filled circles mutant females. A cross line denotes a deceased individual and the arrow indicates proband

DNA sequencing of exons showed a G to A substitution at nucleotide 986 in exon 7 resulting in the change of codon 308 from cysteine to tyrosine in the proband and 8 other family members and relatives (Table 3). A representative sequencing histogram identifying the mutation and the pedigree chart created based on the DNA sequencing results are shown in Fig. 1.

### Scores and FH diagnosis under the DLCN criteria

The total score under the DLCN criteria for each member is presented in Table 4. All 9 individuals with the C308Y mutation had definite FH while the other members that did not have the mutation had unlikely FH.

**Table 4:** The scores and diagnosis under the DLCN criteria

Subject No	Scores and diagnosis						
	Fh	CH	PE	LDL-C levels	MGT	Total scores	Diagnosis
II-9	2	2	6	5	8	23	Definite FH
I-3	2	–	–	5	8	15	Definite FH
I-4	2	–	–	–	–	2	Unlikely FH
III-2	2	–	–	3	8	13	Definite FH
II-6	2	–	–	–	–	2	Unlikely FH
II-7	2	–	–	5	8	15	Definite FH
II-8	2	–	6	3	8	19	Definite FH
II-10	2	–	6	5	8	21	Definite FH
II-11	2	–	–	–	–	2	Unlikely FH
II-1	2	–	–	5	8	15	Definite FH
II-2	2	–	–	–	–	2	Unlikely FH
II-3	2	–	6	5	8	21	Definite FH
II-4	2	–	–	–	–	2	Unlikely FH
II-5	2	–	–	5	8	15	Definite FH
III-1	2	–	–	–	–	2	Unlikely FH

*Fh* family history, *CH* clinical history, *PE* physical examination, *LDL-C* low density lipoprotein-cholesterol, *MGT* molecular genetic testing, *FH* familial hypercholesterolemia

## Discussion

In the past 2 decades about 50 cases of the C308Y mutation have been reported [19, 23–31]. Of these, the vast majority are identified in Chinese living in Taiwan province, Hong Kong and Southeast Asia [19, 23–28, 30, 31]. Using the polymerase chain reaction-single strand conformation polymorphism method, Zhu et al. discovered a case of C308Y in mainland China [19]. In this sole case, however, neither the clinical data nor the examinations of other family members were recorded. In the present study, we employed physical examinations, lipid profiling, DNA sequencing and the DLCN criteria to investigate a proband and his 14 family members and relatives, and found: 1) 9 out of 15 study subjects carried the C308Y mutation in *LDL-R*; 2) apart from the index patient and his father, all other members with the C308Y variant did not have suggestive CHD manifestations; and 3) under the DLCN criteria, all 9 mutants had definite FH.

The C308Y mutation has been reported most often in isolated individuals, and descriptions of the C308Y mutation in multiple family members are rare. Mak et al. discovered the C308Y variant in several first-degree family members in Hong Kong, i.e., a father and his 3 children, but the phenotypic characteristics of the children were not provided [23]. A 12-year old girl in Taiwan, who had a dramatically increased concentration of plasma cholesterol (16.61 mmol/L) and the presence of skin and tendon xanthomas, was identified to be a C308Y mutant [25]. When her family members were consented for molecular genetic testing, the results showed that both of her parents and 3 of her siblings carried the same mutation. In the present study, we genetically analyzed the proband and his 14 family members and relatives, and found 9 individuals carried the C308Y mutation.

The C308Y mutation results in ~ 70% loss of LDL-R activity [25], leading to substantially impaired LDL-C clearance and thereby hypercholesterolemia, which is demonstrated in this study and previous reports as well [19, 23–31]. As pharmacological treatment can effectively lower the concentrations of LDL-C to reduce the risk of early onset CHD [6], all subjects harboring the C308Y mutation should therefore take lipid-lowering pharmacologic agents. In this study, lipid-lowering medications were prescribed to all 9 individuals with definite FH.

There have been over 1700 *LDL-R* variants identified thus far, therefore, discovery of specific fingerprints of *LDL-R* mutations in a region or a population would be helpful for focused cascade screening. The C308Y variant has been revealed to cluster in Taiwan and Hong Kong where the origin of most residents are from Southern China including Guangdong, Hainan and Fu Jian provinces [19, 23–28, 30, 31]; and in the present study we discovered the C308Y mutation in multiple family members in Southern China, leading us to speculate that the C308Y might be a common variant in the population of Southern China origin. This hypothesis however, remains to be validated by screening for the C308Y mutation in a large population and, if confirmed, then a tailored molecular testing of C308Y in this target population might be advisable.

The disease database ClinVar contains the assessment results of the C308Y variant’ pathogenicity (https://www.ncbi.nlm.nih.gov/clinvar/36809953/). According to the evaluation against the American College of Medical Genetics and Genomics (ACMG) guidelines [32], ClinVar classified 3 different pathogenic roles of C308Y mutation, i.e., likely benign, likely pathogenic and pathogenic. These discordant interpretations in pathogenicity indicate that the C308 variant may have different penetrance in different ethnic groups and that environmental factors may affect the pathogenicity of the C308Y variant. The disagreeing pathogenicity documented in ClinVar could also arise from flaw data collected and analyzed by the database, as the ACMG guidelines point out that disease databases may collect variants that are incorrectly classified for analysis as many studies do not describe detailed information on how patients were ascertained [32].

In 2018, a consensus on FH screening, diagnosis and treatment was published in China, which advocates cascade screening if a patient is diagnosed with FH [33]. According to the consensus, cascade screening should include family history, clinical history, physical examination, blood LDL-C measurement and genetic testing [33]. Despite this recommendation, in China cascade screening is hindered mainly by low awareness of FH among patients and physicians as well [34]. With respect to genetic testing, several barriers to the test remain to be overcome, which include a limited number of facilities capable of genetic analysis, patients’ personal fear of genetic diagnosis and the cost of the test that is not covered by the China National Public Health Insurance [34–36].

It has been shown that individuals with the C308Y mutation have hypercholesterolemia but the occurrence of CHD in these patients is not well documented [15, 20–28]. In the present study, apart from the proband and his father, all other individuals with FH did not have clinical manifestations suggestive of CHD, indicating that CHD development is multifactorial.

## Conclusions

The C308Y mutation was discovered in multiple family members and relatives for the first time in mainland China. Cascade screening is key for the confirmatory diagnosis of FH cases with the C308Y mutation. Our hypothesis that the C308Y is a common variant in the population of Southern China origin warrants further validation by screening for the C308Y mutation in a large population.

## Data Availability

All data will be available upon request made to the corresponding author.

## Abbreviations

ACMG: American College of Medical Genetics and Genomics
CHD: Coronary heart disease
DLCN: Dutch Lipid Clinic Network
FH: Familial hypercholesterolemia
LDL-C: Low density lipoprotein-cholesterol
LDL-R: Low density lipoprotein receptor
LOVD: Leiden Open Source Variation Database
